# Dorsal anterior cingulate cortex-targeted transcranial temporal interference stimulation for negative symptoms and cognitive impairment in schizophrenia: a participant- and assessor-blinded, sham-controlled randomized controlled trial protocol

**DOI:** 10.3389/fpsyt.2026.1915709

**Published:** 2026-09-01

**Authors:** Fan Wu, Manling Long, Xin Li, Zixuan Zeng, Sha Ma, Shupei Zhou, Lulu Zhang, Hengfen Gong, Jingjing Huang

**Affiliations:** 1 Clinical Research Center for Mental Disorders, Shanghai Pudong New Area Mental Health Center, School of Medicine, Tongji University, Shanghai, China; 2 School of Medicine, Tongji University, Shanghai, China

**Keywords:** cognition, dACC, negative symptoms (schizophrenia), RCT - randomized controlled trial, schizophrenia, temporal interference stimulation

## Abstract

**Background:**

Persistent negative symptoms and cognitive deficits remain major obstacles to functional recovery in schizophrenia. These impairments are often only partially responsive to antipsychotic treatment and may persist even when other symptoms are clinically stable. Psychosocial interventions show inconsistent efficacy, and commonly used non-invasive brain stimulation techniques have limited ability to target deeper cortical regions. Transcranial temporal interference stimulation (tTIS) has recently emerged as a promising approach for modulating neural activity in such regions. This trial aims to evaluate the clinical efficacy of tTIS targeting the dorsal anterior cingulate cortex (dACC) in individuals with schizophrenia.

**Methods:**

Seventy-four participants with schizophrenia will be enrolled in a parallel-group, sham-controlled trial. After random allocation, half of the participants will receive active stimulation and the remainder will receive sham stimulation. Group assignment will be blinded from participants and clinical evaluators. Each participant will complete 10 sessions over 2 weeks, with one 30-minute session scheduled on each weekday. Assessments will be completed before the first session, after the treatment course, and 4 weeks after treatment completion. Evaluation will include symptom ratings, neuropsychological testing, behavioral tasks, and multimodal neuroimaging. The primary endpoint is the baseline-adjusted between-group difference in the Positive and Negative Syndrome Scale negative symptom subscale (PANSS-NS) score at T1. The PANSS-NS score at T2 will be analyzed as a secondary outcome to evaluate the durability of the treatment effect. The Brief Assessment of Cognition in Schizophrenia composite z-score and the Scale for the Assessment of Negative Symptoms total score are prespecified as key secondary outcomes. Participants will be included in the primary analysis according to their original treatment assignment.

**Discussion:**

This study aims to determine whether dACC-targeted tTIS is feasible and clinically promising in schizophrenia. By combining symptom evaluation with cognitive, behavioral, and imaging measures, the trial may also generate preliminary evidence about treatment-related neural changes. The results will help guide the design of future studies evaluating deep-targeted non-invasive stimulation in psychiatric populations.

**Clinical trial registration:**

## 1 Introduction

Globally affecting approximately 1% of the population, schizophrenia is a recurrent psychiatric disorder associated with significant disability and long-term societal burden (1, 2). Its clinical presentation involves distinct domains, including positive symptoms, negative symptoms, cognitive deficits, and subsequent social and occupational dysfunction. Specifically, early-onset cognitive deficits persist across the lifespan of the illness, acting as a primary barrier to independent living (3). Concurrently, negative symptoms carry substantial prognostic weight, directly correlating with persistent unemployment, premature mortality, and elevated suicide risk. Given that more than half of this patient population experiences at least one clinically significant negative symptom, both cognitive and negative dimensions represent critical targets for therapeutic intervention (4).

Current first-line treatment relies primarily on antipsychotic medication. Most antipsychotics are effective in reducing positive symptoms but have limited effects on negative symptoms and cognitive impairment. Adverse effects, including sedation, metabolic disturbances, and extrapyramidal symptoms, may further compromise treatment adherence and quality of life (5). Psychosocial interventions have also been explored, but the evidence remains inconsistent due to methodological limitations, including small sample sizes, heterogeneity in outcome measures, and variation in follow-up durations (4). Neuromodulation techniques, including electroconvulsive therapy (ECT) and transcranial magnetic stimulation (TMS), provide alternative approaches by modulating brain activity. However, ECT requires general anesthesia and may affect memory, while conventional TMS has a limited ability to precisely target deep brain regions. More targeted and less invasive neuromodulation strategies are therefore needed.

Transcranial temporal interference stimulation (tTIS) achieves non-invasive access to deep neural structures through a distinct biophysical mechanism. Two independent electrode pairs deliver high-frequency alternating currents whose frequencies differ only slightly — for example, by several Hz. Because high-frequency fields alone are largely ineffective at driving neuronal firing, only the region where the two fields spatially overlap experiences a meaningful low-frequency envelope capable of modulating local activity, while tissue closer to the scalp surface remains comparatively unaffected by either carrier frequency in isolation (6). Since the initial report of temporal interference stimulation in 2017, research has progressed from preclinical models to early human studies (7). Animal experiments have investigated its application in modulating epileptic networks (8), and studies involving healthy participants have demonstrated effects on hippocampal memory and working-memory performance (9, 10). Clinical investigations have also examined its potential to improve motor symptoms in Parkinson’s disease (11).

Although tTIS has demonstrated preliminary safety and feasibility in various neurological and psychiatric contexts, its application in schizophrenia remains largely unexamined. Current clinical evidence is limited, with most published studies focusing on the nucleus accumbens (NAc). To date, only one preliminary study and one randomized controlled trial protocol have assessed NAc-targeted tTIS in individuals with schizophrenia (12, 13). While these investigations provide a foundation, the therapeutic potential of alternative brain targets has not been thoroughly explored. The dorsal anterior cingulate cortex (dACC) is a particularly relevant candidate due to its central involvement in cognitive control and motivational processes.

The dACC occupies a central position in the prefrontal–cingulate–striatal network and contributes to several processes relevant to schizophrenia, including performance monitoring, cognitive regulation, and the evaluation of effort in relation to reward (14, 15). Evidence indicates that this region may operate abnormally early in the course of the disorder. Specifically, first-episode schizophrenia has been characterized by higher average controllability but lower modal controllability in the dACC, a pattern that may reflect reduced capacity to flexibly shift between neural states (16). Neurochemical findings provide complementary support for dACC involvement. Magnetic resonance spectroscopy studies have associated local glutamate and glutamine concentrations with cognitive performance in schizophrenia (17). Higher glutamate levels may coincide with diminished local inhibition and reduced inhibitory influence from the dACC to the anterior insula, potentially disrupting processes such as conflict evaluation and evidence accumulation (18). Functional imaging studies further suggest that dACC dysfunction is not limited to established illness. During inhibitory control tasks, reduced bilateral activation has been observed both in patients with schizophrenia and in individuals at clinical high risk. In parallel, the adaptive coupling between the dACC and medial prefrontal cortex that is typically detected in healthy individuals appears to be less prominent in these populations (19).

Interventional evidence suggests that the prefrontal–cingulate–striatal network may have therapeutic value in schizophrenia. Stimulation of the prefrontal cortex using TMS has shown potential benefits for both cognitive deficits and negative symptoms (20). Preliminary findings from deep TMS studies indicate that modulation of the anterior cingulate cortex is well tolerated and may alleviate the overall severity of psychotic symptoms (21). Neuroimaging studies of pharmacological treatment offer converging support for the involvement of this network. Cognitive improvement has been linked to stronger coupling of the dorsolateral prefrontal cortex with the anterior and mid-cingulate regions (22), whereas symptomatic improvement has been related to enhanced connectivity across the striatum, anterior cingulate cortex, and dorsolateral prefrontal cortex (23). Collectively, these observations provide a rationale for evaluating the dACC as a candidate target for neuromodulation in schizophrenia.

Accordingly, we designed a randomized, participant- and outcome-assessor-blinded, sham-controlled trial to evaluate dACC-targeted tTIS in individuals with schizophrenia. The primary objective is to determine whether active stimulation reduces negative symptom severity compared with sham stimulation. Secondary objectives are to examine changes in cognitive performance, broader clinical outcomes, behavioral indices of cognitive control, safety, tolerability, and multimodal neuroimaging measures. The findings may inform the development of more targeted and individualized neuromodulation strategies for schizophrenia.

## 2 Methods and analysis

### 2.1 Trial design

This is a parallel-group clinical trial comparing active and sham tTIS in patients with schizophrenia. Participants who meet the eligibility criteria will be randomly assigned to one of the two conditions in equal proportions. Both participants and investigators responsible for outcome evaluation will remain unaware of treatment allocation. Before enrollment, each participant will provide written informed consent. No trial-specific procedure will be undertaken until consent has been obtained.

**Figure 1:**
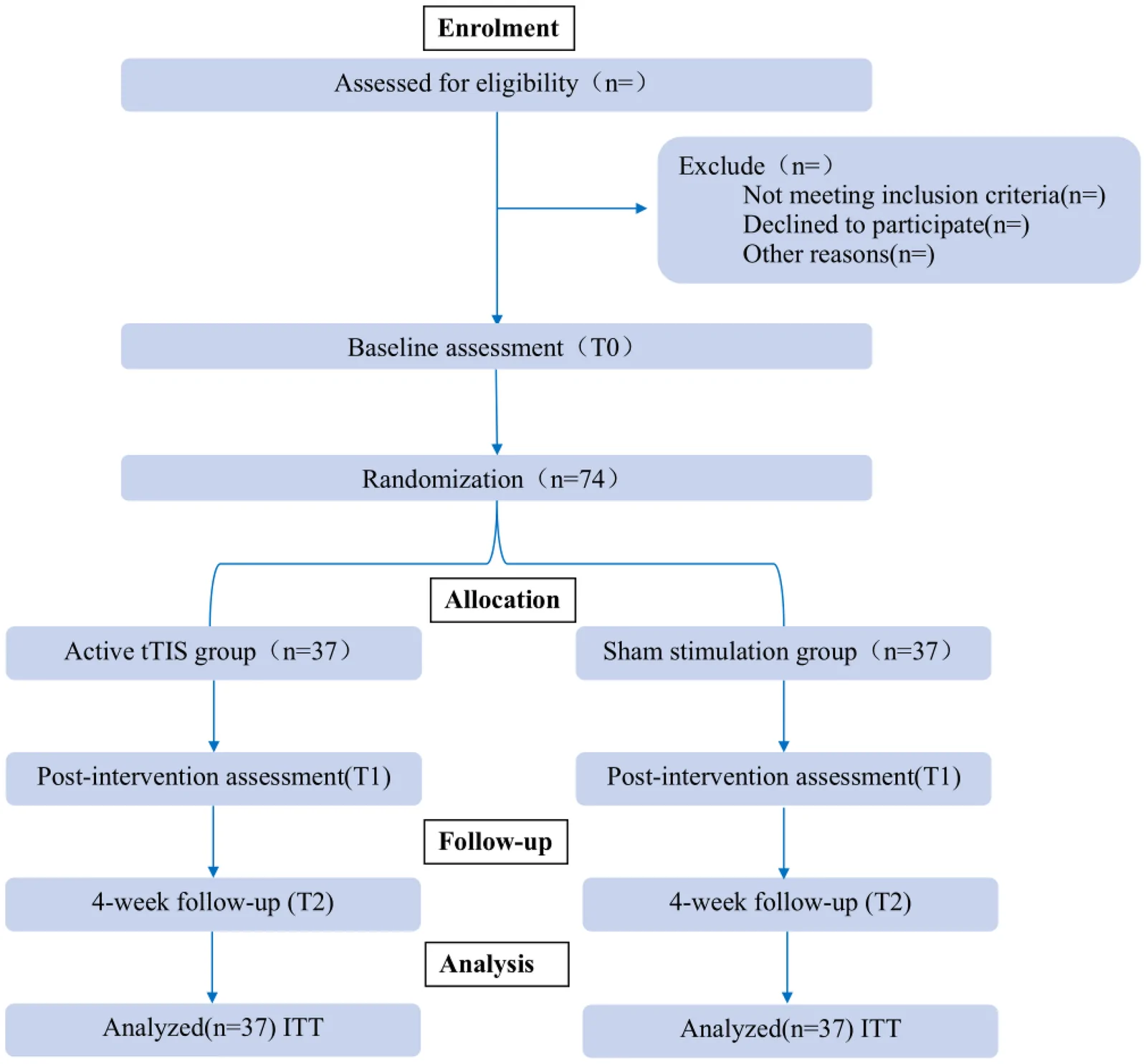
Flow diagram of the study. Adapted from CONSORT 2025 flow diagram published by Hopewell et al. (49) (CC BY 4.0).

Study outcomes will be evaluated at three time points: before the first stimulation session (T0), after completion of the 10-session treatment course (T1), and 4 weeks after the final session (T2). The assessment battery includes clinical ratings, cognitive measures, behavioral tasks, and neuroimaging examinations. **Figure 1** summarizes the sequence of enrollment, intervention, and follow-up procedures.

This protocol was designed following the SPIRIT 2025 recommendations, and findings will be disseminated in accordance with the CONSORT 2025 reporting standards (24, 25). A fully completed SPIRIT 2025 checklist has been included in the **Supplementary Materials**, and **Supplementary Table 1** provides a structured overview of the trial design and methodology, incorporating applicable items from the World Health Organization Trial Registration Data Set. Ethical approval was granted by the Ethics Committee of the Mental Health Center, Tongji University (approval number: PDJW-KY-2025-008GZ-01), and the trial has been prospectively registered on ClinicalTrials.gov (NCT07658898).

#### 2.1.1 Recruitment

Participants will be recruited from both inpatient and outpatient services at Shanghai Pudong Mental Health Center. All participants will be required to meet the same eligibility criteria and to be clinically stable with a stable antipsychotic treatment regimen prior to enrollment. Prior to enrollment, eligibility will be independently assessed by two psychiatrists who are not involved in intervention delivery.

#### 2.1.2 Inclusion criteria

All of the following criteria must be satisfied for enrolment:

1. A confirmed diagnosis of schizophrenia established according to the criteria set forth in the Diagnostic and Statistical Manual of Mental Disorders, Fifth Edition (DSM-5);
2. Age between 18 and 65 years, regardless of sex, with completion of at least junior secondary education and the capacity to undergo neurocognitive testing;
3. Clinically meaningful negative symptoms, operationalized as a PANSS negative symptom subscale (PANSS-NS) total score ≥ 20 with a rating of ≥ 3 on a minimum of one individual PANSS-NS item (26); the PANSS positive symptom subscale score must additionally not exceed 19 (27);
4. Objective cognitive impairment evidenced by an age- and sex-adjusted composite z-score of ≤ −1.0 on the Chinese version of the Brief Assessment of Cognition in Schizophrenia (BACS), reflecting performance at least one standard deviation below normative values derived from Mandarin-speaking populations;
5. Maintenance on an unchanged antipsychotic regimen — with no modification to medication type or dose — for at least 30 days prior to informed consent and continuing throughout the trial;
6. Voluntary agreement to participate, documented by written informed consent following thorough explanation of study objectives and procedures; where applicable, a legally authorized representative may provide or co-sign consent.

#### 2.1.3 Exclusion criteria

Individuals will be ineligible if any of the following conditions apply:

1. A concurrent psychiatric diagnosis requiring active clinical management, including but not limited to major depressive episode or bipolar disorder, or the presence of a comorbid neurological disorder or serious physical illness;
2. Current or past substance use disorder, encompassing problematic consumption of alcohol or illicit substances;
3. A documented seizure disorder or marked electroencephalographic abnormalities, particularly epileptiform activity;
4. Evidence of structural brain pathology, including organic lesions, prior traumatic brain injury, or previous neurosurgical intervention;
5. Presence of ferromagnetic implants or any other contraindication to MRI or tTIS exposure, including intracranial metallic clips, implanted cardiac devices, or cochlear implants;
6. Current use of glucocorticoids or other pharmacological agents known to meaningfully alter cortical excitability;
7. Prior exposure to any neuromodulatory intervention — including modified electroconvulsive therapy (MECT), repetitive transcranial magnetic stimulation (TMS), or transcranial direct current stimulation (tDCS) — within the 30 days preceding enrolment.

### 2.2 Interventions

#### 2.2.1 tTIS device

All tTIS sessions will be delivered using the NervioX-1000 stimulator (Suzhou, China), which integrates a control console, an electrode interface module, preconfigured stimulation protocols, and a scalp cap fitted with disc-shaped electrodes and interconnecting leads. The active surface area of each electrode is 1 cm². Device operation will comply with the manufacturer’s specifications and applicable safety regulations throughout the study.

#### 2.2.2 Active tTIS

Active stimulation will target the dACC using two pairs of scalp electrodes. Individualized electric-field simulations will determine electrode placement, current distribution between channels, and the output intensity for each channel. Based on the role of theta-band activity in dACC-mediated cognitive control and recent human tTIS evidence (28, 29), carrier frequencies of 2000 Hz and 2006 Hz will be used to generate a 6-Hz envelope frequency.

Stimulation intensity will be individualized according to electric-field simulations and participant tolerability. The lowest current sufficient to achieve the prespecified target-region electric field while minimizing off-target exposure will be used. The maximum permitted zero-to-peak current will be 3.6 mA per channel and will serve as an upper limit rather than a fixed treatment dose. Available human tTIS studies have generally reported favorable short-term tolerability, including repeated-session use in psychiatric populations at lower current intensities (12, 13). Because direct evidence for 10 consecutive sessions at the maximum permitted intensity remains limited, stimulation will be closely monitored and reduced, interrupted, or discontinued if clinically significant discomfort, persistent skin irritation, or other safety concerns occur.

Participants will receive 10 sessions over 10 consecutive weekdays, with each session lasting 30 minutes. Current will be ramped up and down over 10 seconds at the beginning and end of each session. Electrode sites and adverse events will be assessed before and after treatment, and stimulation intensity will be reduced, interrupted, or terminated if clinically significant discomfort or safety concerns occur.

#### 2.2.3 Sham tTIS

The sham condition will mirror the active intervention with respect to electrode montage, visit schedule, and session length. To reproduce the transient scalp sensations associated with treatment initiation and termination, brief ramp-up and ramp-down periods will be applied at the beginning and end of each visit. Between these two phases, no continuous current will be delivered.

#### 2.2.4 Individualized optimization of stimulation parameters

To account for interindividual anatomical variability, electrode positioning will be guided by personalized computational modeling rather than a fixed montage. Each participant’s T1-weighted structural scan will first undergo automated tissue segmentation distinguishing scalp, skull, cerebrospinal fluid, and gray and white matter, using SPM12. Standard conductivity values appropriate to each tissue type will be applied, after which a volumetric mesh suitable for finite-element computation will be constructed. The resulting head model will then be used to numerically estimate how the applied currents propagate through cranial tissue, with GetDP performing the underlying field calculations. Candidate electrode positions will be selected from the international 10–10 electroencephalography system. For each candidate configuration consisting of two electrode pairs, the electric fields generated by the two high-frequency carrier currents will be calculated separately. The corresponding temporal interference envelope field will then be derived. The optimal configuration will be selected by considering three criteria: a stronger envelope field within the dACC, greater spatial focality, and lower electric-field exposure in non-target regions.

#### 2.2.5 Criteria for discontinuing or modifying allocated interventions

Further stimulation sessions will not be administered when a participant requests withdrawal, experiences a serious adverse event, or reports ongoing intolerance that cannot be sufficiently managed. Such discomfort may include severe headache, dizziness, or other clinically significant physical symptoms. Treatment cessation may also be considered if a newly identified psychiatric or medical condition renders continuation unsuitable.

Participants who stop receiving stimulation will undergo clinical assessment and will be provided with necessary medical care. The date and circumstances of treatment cessation, together with any associated safety events, will be recorded. Where feasible, these participants will continue to be contacted for the remaining outcome evaluations, irrespective of whether they complete the assigned treatment course. Their available data will be retained in the intention-to-treat analysis.

### 2.3 Outcomes

#### 2.3.1 Primary outcome

The primary endpoint is the baseline-adjusted between-group difference in PANSS-NS score at T1. This subscale consists of seven clinician-rated items, each assigned a value from 1 to 7. The summed score therefore ranges between 7 and 49, with greater values representing more prominent negative symptoms. The Mandarin Chinese PANSS has been psychometrically evaluated, with an internal consistency coefficient of 0.89 reported for its negative symptom component (27).

#### 2.3.2 Key secondary outcomes

Overall neurocognitive functioning will be evaluated using the Mandarin-language adaptation of the BACS, a multidomain battery assessing verbal learning and memory, working memory, psychomotor speed, verbal fluency, sustained attention and processing speed, and executive function. The constituent subtests are the List Learning Test, Digit Sequencing Task, Token Motor Task, Category Instances and Controlled Oral Word Association Test, Symbol Coding Task, and Tower of London Test. Domain scores will be aggregated into a single composite *z*-score, standardized against age- and sex-matched normative data obtained from Mandarin-speaking populations (30), with lower values reflecting greater cognitive impairment. The psychometric adequacy of this adaptation has been established in schizophrenia samples (31).

A more detailed characterization of negative symptomatology will be provided by the Scale for the Assessment of Negative Symptoms (SANS), which comprises 25 clinician-rated items spanning five domains: affective flattening or blunting, alogia, avolition–apathy, anhedonia–asociality, and attentional impairment. Each item is rated on a six-point scale from 0 to 5, and higher summed scores denote a more severe negative symptom profile (32).

#### 2.3.3 Other secondary outcomes

##### 2.3.3.1 Clinical symptoms

The PANSS-NS score assessed at T2 will be analyzed as a secondary outcome to evaluate the durability of the treatment effect.

Broader psychopathological status will be captured using the full 30-item Positive and Negative Syndrome Scale (PANSS), in which clinician-assigned ratings across positive, negative, and general psychopathology subscales are each anchored on a 1–7 ordinal scale (33). Given that the PANSS-NS subscale serves as the primary outcome, secondary analyses will examine the PANSS total score alongside the positive symptom and general psychopathology subscale scores.

##### 2.3.3.2 Apathy and pleasure experience

Apathy will be measured with the self-rated Apathy Evaluation Scale (AES-S), first developed by Marin and colleagues (34). The scale’s 18 items probe the cognitive, affective, and behavioral dimensions of apathetic experiences over the preceding four weeks, yielding a total score between 18 and 72. The Chinese adaptation has demonstrated satisfactory internal consistency (Cronbach’s α = 0.89) (35).

Hedonic experience will be evaluated using the Temporal Experience of Pleasure Scale (TEPS), developed by Gard and colleagues (36), which separately indexes anticipatory and consummatory components of pleasure across 20 items. Each item is rated on a six-point Likert scale (1 = “very false for me” to 6 = “very true for me”); following reverse-scoring of designated items, responses are summed to yield a total score, with lower values indicating greater anhedonia. Psychometric data from Chinese-speaking populations confirm the suitability of the translated scale.

##### 2.3.3.3 Social functioning and quality of life

Real-world social functioning will be evaluated by a trained clinician using the Personal and Social Performance Scale (PSP), which appraises four behavioral domains: engagement in socially constructive activities, interpersonal relationships, self-care, and disruptive or aggressive conduct. Scores span 1 to 100, with higher scores reflecting superior functional status; its utility in Chinese schizophrenia populations has been previously documented (37).

Subjective quality of life will be assessed using the World Health Organization Quality of Life-BREF (WHOQOL-BREF), a multidimensional instrument covering four domains: physical health, psychological well-being, social relationships, and environment. Higher scores within each domain reflect a more positive self-reported quality of life. The Chinese adaptation has undergone formal validation in mainland Chinese samples (38).

##### 2.3.3.4 Sleep and emotional symptoms

Sleep disturbance will be quantified with the Pittsburgh Sleep Quality Index (PSQI), a 19-item self-report measure that generates a composite global score from 0 to 21, where higher values indicate worse sleep quality. Satisfactory reliability and validity of the Chinese adaptation have been reported in prior studies (39).

Current depressive symptomatology will be quantified using the Patient Health Questionnaire-9 (PHQ-9), in which nine items — each rated 0 to 3 — yield an aggregate score of 0 to 27; elevated scores reflect more severe depressive burden. The Chinese version has demonstrated strong internal consistency (Cronbach’s α = 0.86) (40).

Anxiety over the preceding two weeks will be indexed by the Generalized Anxiety Disorder-7 scale (GAD-7), comprising seven items each scored 0–3 and summed to a maximum of 21; higher totals denote greater anxiety severity. For the Chinese adaptation, internal consistency (Cronbach’s α = 0.898) and test–retest reliability (r = 0.856) have been established (41).

##### 2.3.3.5 Behavioral measures of cognitive control

Given the central role of the dACC in cognitive control, participants will complete the Go/No-Go and Stroop color–word tasks. Response inhibition will be evaluated with the Go/No-Go paradigm, in which participants are required to respond promptly to Go stimuli while withholding responses to No-Go stimuli (42). The derived indices will comprise overall accuracy, Go and No-Go accuracy, omission and commission error rates, reaction time for correct Go trials, and *d*′. The Stroop color–word task will be administered to characterize conflict processing and cognitive control. Participants will identify the font color of each stimulus while disregarding the meaning of the displayed word (43). The analysis will include overall and condition-specific accuracy, reaction times for congruent and incongruent trials, and the Stroop interference effect.

##### 2.3.3.6 Neuroimaging outcomes

MRI data will be acquired on a 3.0-T uMR 780 scanner (United Imaging Healthcare, Shanghai, China). Exploratory analyses will include rs-fMRI and DTI measures.

DTI will be collected with 64 diffusion directions and b-values of 0 and 1000 s/mm². Whole-brain coverage will be obtained with 64 interleaved slices at 2.5-mm isotropic resolution. The sequence parameters will be TR = 10,232 ms, TE = 69.7 ms, flip angle = 90°, field of view = 220 × 220 mm², and anterior-to-posterior phase encoding. For rs-fMRI, echo-planar images will be acquired with 37 interleaved slices and a voxel size of 3 × 3 × 3.5 mm³. The sequence will use TR = 2000 ms, TE = 30 ms, flip angle = 90°, field of view = 216 × 216 mm², and posterior-to-anterior phase encoding. Other assessments and covariates.

Baseline data collection will cover demographic characteristics, clinical history, and lifestyle-related factors. Demographic variables will include age, sex, marital status, educational attainment, employment status, and income level. Clinical information will include illness duration, age at onset, previous hospitalizations, treatment history, and the type and dosage of current antipsychotic medication. Smoking status, alcohol consumption, and other potentially relevant lifestyle factors will also be recorded. Antipsychotic doses will be converted into daily chlorpromazine-equivalent doses using the international consensus method proposed by Gardner et al. For participants receiving more than one antipsychotic medication, the chlorpromazine-equivalent dose of each agent will be calculated separately and then summed to obtain the total daily chlorpromazine-equivalent dose. These values will be summarized descriptively and, when prespecified, incorporated into covariate-adjusted analyses.

Participants’ perceptions of the credibility of tTIS and their expectations regarding treatment benefit will be measured at baseline with an adapted version of the Credibility/Expectancy Questionnaire (CEQ) (44). CEQ scores will be considered as potential covariates in sensitivity analyses.

### 2.4 Safety assessments

The incidence and severity of adverse events will be assessed throughout the study period, from baseline to the 4-week follow-up. Treatment tolerability and adherence will also be documented during the intervention period. Detailed procedures for adverse event monitoring and reporting are described in the section on oversight and monitoring.

### 2.5 Sample size

Sample size was estimated using a Monte Carlo simulation–based power analysis for a mixed-effects model for repeated measures (MMRM). Based on findings from previous meta-analyses of non-invasive brain stimulation for negative symptoms in schizophrenia, the anticipated standardized between-group effect size at T1 was set at Cohen’s (d=0.60) (45). To account for a possible partial attenuation of the treatment effect during follow-up, the effect size at T2 was conservatively set at (d=0.40).

The correlations between baseline and T1, baseline and T2, and T1 and T2 assessments were assumed to be 0.50, 0.40, and 0.60, respectively. The cumulative rates of missing outcome data were assumed to be 5% at T1 and 10% at T2. The MMRM used in the simulations included treatment group, visit, the treatment group-by-visit interaction, baseline PANSS-NS score, and the baseline score-by-visit interaction as fixed effects. An unstructured covariance matrix, restricted maximum likelihood estimation, and the Kenward–Roger approximation were applied.

Simulations were conducted in R version 4.6.1 using the MASS, mmrm version 0.3.18, and emmeans version 2.0.4 packages. For each candidate sample size, 10,000 simulated trials were generated. Statistical power was defined as the proportion of simulations in which the two-sided (P) value for the primary between-group comparison at T1 was less than 0.05. The estimated power was 79.86% with 36 participants per group and 80.77% with 37 participants per group. Therefore, the planned sample size is 74 participants, with 37 participants allocated to each group.

### 2.6 Assignment of interventions

#### 2.6.1 Sequence generation

Randomization will take place after the baseline evaluation has been completed and eligibility has been confirmed. Participants will enter either the active tTIS arm or the sham arm with equal probability. The allocation list will be created in IBM SPSS Statistics version 25.0 using a simple randomization procedure. This task will be undertaken by a researcher whose responsibilities are restricted to randomization management and who will take no part in recruitment, eligibility screening, stimulation delivery, outcome evaluation, or data analysis.

#### 2.6.2 Allocation concealment

Group assignments will be concealed in consecutively numbered, non-transparent, sealed envelopes with the same external appearance. The envelopes will be assembled and retained by the researcher responsible for randomization management. Once a participant has completed the baseline evaluation and enrollment procedures, the intervention operator will open the next available envelope immediately before treatment allocation is implemented. The allocation list will remain unavailable to outcome assessors and to research personnel who do not require access for intervention delivery.

#### 2.6.3 Blinding

Participants and outcome assessors will be blinded to treatment allocation. The active and sham stimulation procedures will follow the same schedule and use identical electrode placement procedures. The sham stimulation procedure will include ramp-up and ramp-down phases to mimic the sensory experience of active stimulation. Because the intervention parameters must be configured according to group allocation, the staff member responsible for delivering the stimulation cannot be blinded. This staff member will also be responsible for safety monitoring and adverse-event assessments during the intervention period, but will not participate in participant recruitment, clinical or cognitive assessments, data management, or statistical analysis. Safety and adverse-event information will be recorded separately and will not be disclosed to the blinded outcome assessors. Interactions between the intervention staff and participants will be limited to those necessary for stimulation delivery and safety monitoring.

The researchers responsible for data analysis will remain blinded to group allocation until the primary analyses have been completed. Group labels will be coded using neutral identifiers, such as Group A and Group B. The allocation code will be revealed only after the statistical analysis plan has been finalized and the main analyses have been completed.

After the intervention, participants and outcome assessors will be asked to guess the assigned treatment group and indicate the basis for their judgment.

### 2.7 Data collection and management

Outcome evaluations will be completed at T0, T1, and T2. **Table 1** provides a detailed overview of participant enrollment, treatment delivery, and data collection across the study period.

**Table 1:** SPIRIT schedule of enrollment, interventions, and assessments.

Study phase/assessment	Screening	Baseline and randomization (T0)	Intervention period (Weeks 1–2)	Post-intervention (T1)	4-week follow-up (T2)
Enrolment and allocation					
Eligibility screening	x				
Informed consent	x				
Demographic and clinical characteristics		x			
Randomization		x			
CEQ		x			
Interventions					
Active TI stimulation			10 sessions, 30 min/session		
Sham stimulation			10 sessions, 30 min/session		
Outcome assessments					
PANSS-NS		x		x	X
BACS composite *z* score		x		x	X
SANS score		x		x	X
Other secondary clinical outcomes^a^		x		x	X
Go/No-Go and Stroop tasks		x		x	X
Structural MRI (T1-weighted)		x			
rs-fMRI and DTI		x		x	X
Safety, tolerability, treatment adherence, and adverse events		x	x	x	X

BACS, Brief Assessment of Cognition in Schizophrenia; CEQ, Credibility/Expectancy Questionnaire; DTI, diffusion tensor imaging; PANSS, Positive and Negative Syndrome Scale; PANSS-NS, PANSS negative symptom subscale; rs-fMRI, resting-state functional magnetic resonance imaging; TI, temporal interference stimulation.

^a^Other secondary clinical outcomes include PANSS total, positive symptom subscale, and general psychopathology subscale scores; self-rated Apathy Evaluation Scale (AES-S); Temporal Experience of Pleasure Scale (TEPS); Personal and Social Performance Scale (PSP); World Health Organization Quality of Life-BREF (WHOQOL-BREF); Pittsburgh Sleep Quality Index (PSQI); Patient Health Questionnaire-9 (PHQ-9); and Generalized Anxiety Disorder-7 (GAD-7).

Study records will be handled in accordance with the purposes of the trial. Upon enrollment, each participant will be identified by a study-specific code. Information that could reveal the identity of an individual participant will be maintained separately from the analysis dataset and will not appear in research reports, conference materials, or publications. Data will initially be recorded on paper case report forms (CRFs). Two data managers will independently transfer the information into a password-protected electronic database. The two electronic records will then be compared. Where inconsistencies are identified, the original CRFs will be reviewed, with source documents consulted when clarification is required.

Before enrollment commences, all personnel involved in the study will complete protocol-specific training appropriate to their responsibilities. Clinical assessors will be trained in the consistent administration of symptom ratings and cognitive measures, while intervention operators will receive instruction in the standardized delivery of tTIS and the recording of adverse events. MRI technicians will be briefed on the acquisition protocol and image-quality requirements. Training will also address participant communication and the accurate completion of study documentation.

### 2.8 Statistical methods

Statistical analyses will be conducted in IBM SPSS Statistics version 25.0 and R version 4.6.1. Categorical data will be described using counts and proportions. For continuous variables, means with standard deviations will be reported when the distribution is approximately symmetric; otherwise, medians with interquartile ranges will be provided. Baseline characteristics will be summarized separately for the two treatment arms. All inferential tests will be two-tailed. Results will be reported with effect estimates and 95% confidence intervals. The primary efficacy test will use a two-sided significance level of α=0.05, whereas P values for secondary and exploratory analyses will be considered nominal unless otherwise specified.

For repeatedly measured post-randomization outcomes, the primary analytical framework will be an MMRM. Outcome measurements at T1 and T2 will constitute the repeated dependent observations, with visit modeled as a categorical variable. The model will include treatment group, visit, the treatment group-by-visit interaction, the baseline value of the corresponding outcome, and the baseline value-by-visit interaction as fixed effects. Within-participant correlations will be modeled using an unstructured covariance matrix. Parameters will be estimated using restricted maximum likelihood, with the Kenward–Roger method used to adjust the covariance matrix of the fixed-effect estimates and denominator degrees of freedom. Baseline-adjusted between-group contrasts will be estimated separately at T1 and T2. To facilitate clinical interpretation, descriptive within-group changes from baseline to T1 and T2 will also be reported for each treatment group, including mean changes and corresponding 95% confidence intervals.

#### 2.8.1 Primary outcome analysis

The primary analysis will follow the intention-to-treat principle and include all randomized participants according to their assigned treatment group. The prespecified MMRM will be applied to PANSS-NS scores at T1 and T2. The baseline-adjusted between-group contrast at T1 will constitute the sole primary efficacy test and will be reported as an adjusted mean difference with a 95% confidence interval and two-sided P value. The corresponding contrast at T2 will be considered secondary and used to assess the durability of the treatment effect.

#### 2.8.2 Key secondary outcome analyses

The BACS composite z-score and the SANS total score are designated as key secondary outcomes. Each measure will be examined separately using the same longitudinal modeling strategy as the primary endpoint. Adjusted group contrasts at T1 and T2 will be used to characterize changes immediately after the intervention and at the 4-week follow-up. As these outcomes are intended to provide supportive evidence, interpretation will focus on effect sizes, confidence intervals, and the consistency of findings across assessment points.

#### 2.8.3 Other secondary outcome analyses

Additional secondary outcomes include the PANSS total score, PANSS positive symptom subscale score, PANSS general psychopathology subscale score, AES-S, TEPS, PSP, WHOQOL-BREF, PSQI, PHQ-9, and GAD-7 scores, as well as indices derived from the Go/No-Go and Stroop tasks. Repeatedly collected continuous measures will be analyzed using the prespecified MMRM framework. These analyses are exploratory and will be interpreted primarily in terms of estimated effect magnitude and precision.

#### 2.8.4 Missing data and sensitivity analyses

The frequency, timing, and documented causes of missing observations will be summarized. The mixed-effects models will incorporate all available outcome data under a missing-at-random assumption. Robustness of the primary findings will be examined in a per-protocol analysis. The per-protocol population will include participants who complete at least 80% of the scheduled intervention sessions and have no major protocol deviations. Further sensitivity models will additionally adjust for clinically relevant baseline covariates, including age, sex, illness duration, chlorpromazine-equivalent antipsychotic dose, and baseline treatment expectancy. Given the modest sample size, treatment-by-covariate interaction analyses will be limited; any analysis examining illness duration as a potential modifier of treatment effect will be considered exploratory.

#### 2.8.5 Exploratory neuroimaging analyses

Neuroimaging data will be acquired at T0, T1, and T2 and analyzed as exploratory outcomes. Imaging analyses will primarily focus on the prefrontal–cingulate–striatal circuit. All cortical and subcortical regions of interest (ROIs) will be defined according to the Brainnetome Atlas. The dACC will serve as the core ROI and will be operationally defined as the combined bilateral caudodorsal area 24 (A24cd) and pregenual area 32 (A32p) parcels in the Brainnetome Atlas. Additional prespecified ROIs will include the bilateral dorsolateral prefrontal cortex, anterior insula, caudate nucleus, putamen, and nucleus accumbens. The same atlas version, spatial masks, and metric-extraction procedures will be applied consistently across all participants and imaging time points.

Resting-state functional magnetic resonance imaging data will be preprocessed using DPABI and SPM12. Preprocessing will include removal of initial volumes, slice-timing correction, head-motion correction, susceptibility-distortion correction based on dual-echo field maps, functional-to-structural image registration, tissue segmentation, normalization to Montreal Neurological Institute space, nuisance-signal regression, detrending, temporal filtering, and spatial smoothing. The SPM12 FieldMap toolbox will be used for field-map correction. DPABI will be used to calculate the fractional amplitude of low-frequency fluctuations, regional homogeneity, ROI-to-ROI functional connectivity, and whole-brain seed-based functional connectivity using the dACC as the seed region.

Diffusion-weighted imaging data will be processed using FSL. Susceptibility-induced distortions will be corrected with TOPUP using b0 images acquired with anterior-to-posterior and posterior-to-anterior phase encoding, and EDDY will be used to correct for eddy-current distortions, head motion, and outlier signals. Brain extraction will subsequently be performed using BET, and DTIFIT will be used to generate fractional anisotropy, mean diffusivity, axial diffusivity, and radial diffusivity maps. Metrics for prespecified white-matter tracts will be extracted according to the JHU white-matter atlas. Whole-brain diffusion analyses will be conducted using tract-based spatial statistics and Randomise, with permutation testing and threshold-free cluster enhancement applied to control for multiple comparisons across the whole brain.

Longitudinal analyses of prespecified ROI-based imaging outcomes will be conducted using the MMRM framework described above. Post-baseline imaging measures at T1 and T2 will constitute the repeated dependent observations, with visit modeled as a categorical variable. Each model will include treatment group, visit, the treatment group-by-visit interaction, the corresponding baseline imaging value, and the baseline value-by-visit interaction as fixed effects. Within-participant correlations will be modeled using an unstructured covariance matrix, and parameters will be estimated using restricted maximum likelihood with the Kenward–Roger adjustment. Prespecified contrasts will estimate baseline-adjusted between-group differences at T1 and T2. False discovery rate correction will be applied across prespecified ROI-based tests. Whole-brain voxelwise analyses will be conducted separately using mass-univariate general linear models and permutation-based inference, with appropriate correction for multiple comparisons. These analyses will be considered secondary and hypothesis-generating. Correlation and regression analyses will explore associations between imaging changes and changes in clinical, cognitive, and behavioral outcomes.

#### 2.8.6 Safety analysis

Safety data will be presented descriptively, stratified by allocated treatment arm. For each adverse event type, the absolute count and percentage of affected participants will be reported, alongside characterization of event onset, duration, severity grade, clinical management, resolution status, and putative causal relationship to the study intervention.

### 2.9 Plans for sharing the protocol, participant-level data, and statistical code

Upon publication of the primary results, the full study protocol and pre-specified statistical analysis plan will be made available to qualified investigators upon reasonable written request to the corresponding author, subject to applicable ethical and data governance requirements.

### 2.10 Oversight and monitoring

#### 2.10.1 Composition of the data and safety monitoring board, its role, and reporting structure

An independent Data and Safety Monitoring Board (DSMB) will be established to provide ongoing oversight of participant welfare and trial conduct. The DSMB will consist of two psychiatrists, a biostatistician, and a specialist in research ethics. To preserve independence, no board member will be involved in any aspect of participant recruitment, intervention delivery, outcome assessment, or data analysis, and all members must be free of financial or professional conflicts of interest that could compromise their objectivity.

The DSMB will convene at predefined intervals to review enrollment progress, participant withdrawals, protocol deviations, and both non-serious and serious adverse events. Unscheduled meetings may be called at any time should emergent safety signals warrant immediate review. After each meeting, the board may issue one of the following recommendations: continuation without modification, protocol amendment, temporary suspension of enrollment, or early trial termination. All formal recommendations will be transmitted to the principal investigator and, where indicated, to the Ethics Committee of the affiliated Mental Health Center, Tongji University.

#### 2.10.2 Adverse event reporting and harms

Every AE occurring from enrollment through the end of the follow-up period will be prospectively recorded in CRFs, with documentation encompassing event description, date of onset, duration, severity, therapeutic measures taken, clinical outcome, and the assigned investigator’s assessment of its relationship to tTIS. A designated study physician will adjudicate potential attribution to the intervention for each event. Non-serious AEs will receive clinically appropriate management and be tracked until full resolution or stabilization. Any SAE must be reported to the principal investigator within 24 hours of the research team becoming aware, and subsequently notified to the Ethics Committee in accordance with institutional and regulatory obligations. AEs judged as possibly or probably related to tTIS will be escalated to the DSMB, which will deliberate on whether additional safety measures, protocol modifications, temporary recruitment suspension, or premature trial closure are warranted.

#### 2.10.3 Monitoring of trial conduct

The research team will perform regular checks of recruitment procedures, informed consent, randomization, intervention delivery, outcome evaluation, and data entry. Protocol deviations will be documented with details of their causes, severity, and possible implications for participant safety and trial integrity. Protocol revisions will not be implemented until Ethics Committee approval has been obtained. Where an amendment may affect participant rights, safety, or continued participation, the relevant information will be communicated to participants and written consent will be renewed as appropriate. Measures needed to address an immediate safety concern may be introduced without prior approval, provided that they are reported to the Ethics Committee without delay.

#### 2.10.4 Dissemination plans

The results of the study will be shared through journal articles and presentations at scientific meetings. Results will be reported transparently and in full, irrespective of their direction or statistical significance.

## 3 Discussion

The present trial evaluates the effects of dACC-targeted tTIS relative to a sham condition in individuals diagnosed with schizophrenia. The primary objective is to determine whether active stimulation reduces negative symptom severity, as reflected by the baseline-adjusted between-group difference in PANSS-NS scores at T1. The BACS composite z-score and SANS total score are designated as key secondary outcomes, assessing overall cognitive performance and a broader range of negative symptom dimensions, respectively. The PANSS-NS score at T2 will be examined as a secondary outcome to assess the durability of the treatment effect. Behavioral measures of cognitive control and multimodal neuroimaging outcomes will also be explored to characterize treatment-related functional and structural changes and generate hypotheses regarding the neural mechanisms of tTIS.

A key feature of this protocol is the selection of the dACC as the stimulation target. Existing tTIS research in schizophrenia has mainly focused on the nucleus accumbens (13). Unlike previous approaches, this study directs stimulation toward the dACC, a region that contributes to performance monitoring, cognitive regulation, and the evaluation of effort relative to reward. Targeting this node may broaden the therapeutic options for negative symptoms and cognitive deficits in schizophrenia. In addition, stimulation parameters will be optimized individually. Structural MRI data will be used to construct subject-specific head models, and electric-field simulations will be applied to optimize electrode locations and current parameters. This approach may reduce variability in target engagement caused by individual differences in head anatomy (46). The combination of clinical, cognitive, behavioral, and neuroimaging measures will allow a broader evaluation of both treatment effects and potential neural mechanisms.

This study also has several limitations. First, because of the operational characteristics of the current tTIS device, the staff responsible for intervention delivery cannot be blinded to treatment allocation. To reduce the risk of performance bias, intervention delivery, outcome assessment, and statistical analysis will be performed by separate personnel. Outcome assessors and data analysts will remain blinded, and interactions between intervention staff and participants will be limited to those required for treatment delivery (25). Second, differences in scalp sensations may affect the effectiveness of the sham procedure. Although brief ramp-up and ramp-down phases will be used to mimic the sensory experience of active stimulation, this approach may not completely mask group allocation in all participants (47). Treatment expectancy will therefore be assessed at baseline, and blinding success will be evaluated after the intervention. Third, the 10-session intervention period and the 4-week follow-up are relatively short for evaluating changes in negative symptoms. The study may therefore underestimate the longer-term therapeutic potential of dACC-targeted tTIS. Fourth, the exploratory nature of this single-center study and its modest sample size limit the broader applicability of the findings. Larger multicenter trials with longer treatment courses and extended follow-up periods will be required to evaluate the durability and generalizability of the observed effects. The neuroimaging analyses are also exploratory and may be influenced by antipsychotic dose, illness duration, baseline symptom severity, and individual variability (48).

This study will generate initial evidence regarding the clinical relevance of dACC-focused tTIS for schizophrenia and its associated neurobiological changes. The integration of symptom ratings, cognitive measures, behavioral performance, and multimodal imaging may help clarify whether modulation of this circuit is feasible and therapeutically promising. The results will provide a foundation for subsequent multicenter studies with larger samples and may contribute to the refinement of individualized, circuit-informed neuromodulation approaches for schizophrenia.

## Trial status

This protocol was registered on ClinicalTrials.gov on June 18, 2026 (NCT07658898). Participant enrollment began on June 30, 2026 and is expected to be completed by January 1, 2027. The study period, including the intervention and follow-up phases for all participants, is expected to extend through December 30, 2027.
