# Supplementary material for: Dorsal anterior cingulate cortex-targeted transcranial temporal interference stimulation for negative symptoms and cognitive impairment in schizophrenia: a participant- and assessor-blinded, sham-controlled randomized controlled trial protocol
Source: Front Psychiatry. 2026 Sep 1;17:1915709. doi: 10.3389/fpsyt.2026.1915709 (PMC13574950; doi:10.3389/fpsyt.2026.1915709)
Supplement: Supplementary file 1 [file Table1.docx]

Supplementary Material

# 1 Supplementary Tables

**Table 1. Structured summary of trial design and methods, including items from the World Health Organization Trial Registration Data Set.**

| **WHO Trial Registration Data Set item** | **Description** |
| --- | --- |
| **Primary registry and trial identifying number** | ClinicalTrials.gov (Registration ID: NCT07658898) |
| **Date of registration in primary registry** | 2026/6/18 |
| **Secondary identifying numbers** | Ethics approval number: PDJW-KY-2025-008GZ-01; protocol version: Version 03, [2026/1/6] |
| **Source(s) of monetary or material support** | PKJ2024-Y87、PKJ2025-Y26、2025-PWXZ-08、PWRL2024-06; material support: the tTIS device is purchased by the research team |
| **Primary sponsor** | Shanghai Pudong New Area Mental Health Center, Shanghai, China |
| **Secondary sponsor(s)** | None |
| **Contact for public queries** | Hengfen Gong, [gonghf@shspdjw.com](mailto:gonghf@shspdjw.com)，[021-68306699](tel:02168306699)，No. 165 Sanlin Road, Pudong New Area, Shanghai 200124, China |
| **Contact for scientific queries** | Jingjing Huang, Vice Dean, Shanghai Pudong New Area Mental Health Center, [huangjingjing18@tongji.edu.cn，021-68306699*1222](mailto:huangjingjing18@tongji.edu.cn,%20021-68306699*1222) ,No. 165 Sanlin Road, Pudong New Area, Shanghai 200124, China |
| **Public title** | A study of temporal interference stimulation for improving negative symptoms and cognitive impairment in people with schizophrenia |
| **Scientific title** | Dorsal Anterior Cingulate Cortex-Targeted Transcranial Temporal Interference Stimulation for Negative Symptoms and Cognitive Impairment in Schizophrenia: A Participant- and Assessor-Blinded, Sham-Controlled Randomized Controlled Trial Protocol |
| **Countries of recruitment** | China |
| **Health condition(s) or problem(s) studied** | Schizophrenia |
| **Intervention(s)** | **Active tTIS group:** Participants will receive individualized dorsal anterior cingulate cortex-targeted transcranial temporal interference stimulation. Two pairs of scalp electrodes will deliver alternating currents at 2000 Hz and 2006 Hz, generating a 6-Hz envelope frequency. Stimulation will be administered for 30 minutes per session over 10 consecutive weekdays. **Sham stimulation group:** Participants will undergo identical electrode placement and treatment schedules, including brief ramp-up and ramp-down phases, but no sustained stimulation will be delivered during the remaining intervention period. |
| **Key inclusion and exclusion criteria** | **Inclusion criteria:** Adults aged 18–65 years who meet DSM-5 criteria for schizophrenia; have clinically significant negative symptoms; have cognitive impairment defined by an age- and sex-adjusted Brief Assessment of Cognition in Schizophrenia composite z-score of ≤ −1.0; and have received stable antipsychotic treatment during the previous 30 days. **Exclusion criteria:** Other major psychiatric disorders, neurological disorders or severe medical conditions; substance abuse or dependence; history of seizures; organic brain lesions; contraindications to magnetic resonance imaging or transcranial temporal interference stimulation; and receipt of brain stimulation treatment within the previous 30 days. |
| **Study type** | Interventional, exploratory, single-centre, randomized, parallel-group, participant- and outcome-assessor-blinded, sham-controlled clinical trial. Participants and outcome assessors will be blinded to treatment allocation. |
| **Date of first enrolment** | 2026/6/30 |
| **Target sample size** | 74 participants |
| **Recruitment status** | Recruiting |
| **Primary outcome(s)** | **Negative symptom severity:** change in the Positive and Negative Syndrome Scale negative symptom subscale score from baseline to immediately after the 10-session intervention. |
| **Key secondary outcome(s)** | Overall psychiatric symptoms, negative symptoms, the Brief Assessment of Cognition in Schizophrenia, apathy, pleasure experience, social functioning, quality of life, sleep quality, depressive symptoms, anxiety symptoms, behavioral measures derived from the Go/No-Go and Stroop tasks, safety outcomes, tolerability outcomes, treatment adherence, and exploratory neuroimaging outcomes. Assessments will be conducted at baseline, immediately after the intervention, and at the 4-week follow-up, except where otherwise specified. |
| **Ethics review** | Approved by the Ethics Committee of the Mental Health Center affiliated with Tongji University. Approval number: PDJW-KY-2025-008GZ-01. Approval date: 2026/3/9. |
| **Completion date** | 2027/12/30 |
| **Summary results** | Not applicable at the protocol stage. Results will be reported after trial completion. |
| **Individual participant data sharing statement** | De-identified participant-level data may be made available from the corresponding author upon reasonable request, subject to approval by the relevant ethics committee and compliance with applicable data protection requirements. |

**Table 2. SPIRIT 2025 checklist of items to address in a randomized trial protocol**

| **Section / Topic** | **No** | **SPIRIT 2025 checklist item description** | **Location in manuscript** |
| --- | --- | --- | --- |
| **Administrative information** | | |  |
| Title and structured summary | 1a | Title stating the trial design, population, and interventions, with identification as a protocol | Title |
|  | 1b | Structured summary of trial design and methods, including items from the World Health Organization Trial Registration Data Set | Abstract; Supplementary Table 1 |
| Protocol version | 2 | Version date and identifier | Supplementary Table 1 |
| Roles and responsibilities | 3a | Names, affiliations, and roles of protocol contributors | Title page; Author contributions |
|  | 3b | Name and contact information for the trial sponsor | N/A |
|  | 3c | Role of trial sponsor and funders in design, conduct, analysis, and reporting of trial; including any authority over these activities | N/A |
|  | 3d | Composition, roles, and responsibilities of the coordinating site, steering committee, endpoint adjudication committee, data management team, and other individuals or groups overseeing the trial, if applicable | Section 2.7, Data collection and management; Section 2.10, Oversight and monitoring |
| **Open science** | | |  |
| Trial registration | 4 | Name of trial registry, identifying number (with URL), and date of registration. If not yet registered, name of intended registry | Abstract (Clinical trial registration); Section 2.1, Trial design; Trial status |
| Protocol and statistical analysis plan | 5 | Where the trial protocol and statistical analysis plan can be accessed | Section 2.9, Plans for sharing the protocol, participant-level data, and statistical code |
| Data sharing | 6 | Where and how the individual de-identified participant data (including data dictionary), statistical code, and any other materials will be accessible | Section 2.9, Plans for sharing the protocol, participant-level data, and statistical code; Data availability statement |
| Funding and conflicts of interest | 7a | Sources of funding and other support (e.g., supply of drugs) | Funding |
|  | 7b | Financial and other conflicts of interest for principal investigators and steering committee members | Conflict of interest statement |
| Dissemination policy | 8 | Plans to communicate trial results to participants, healthcare professionals, the public, and other relevant groups (e.g., reporting in trial registry, plain language summary, publication) | Section 2.10.4, Dissemination plans |
| **Introduction** | | |  |
| Background and rationale | 9a | Scientific background and rationale, including summary of relevant studies (published and unpublished) examining benefits and harms for each intervention | Section 1, Introduction |
|  | 9b | Explanation for choice of comparator | Section 2.2.3, Sham tTIS |
| Objectives | 10 | Specific objectives related to benefits and harms | Abstract; Section 1, Introduction (final paragraph) |
| **Methods: Patient and public involvement, trial design** | | |  |
| Patient and public involvement | 11 | Details of, or plans for, patient or public involvement in the design, conduct, and reporting of the trial | N/A |
| Trial design | 12 | Description of trial design including type of trial (e.g., parallel group, crossover), allocation ratio, and framework (e.g., superiority, equivalence, non-inferiority, exploratory) | Section 2.1, Trial design |
| **Methods: Participants, interventions, and outcomes** | | |  |
| Trial setting | 13 | Settings (e.g., community, hospital) and locations (e.g., countries, sites) where the trial will be conducted | Section 2.1.1, Recruitment |
| Eligibility criteria | 14a | Eligibility criteria for participants | Sections 2.1.2–2.1.3, Inclusion and exclusion criteria |
|  | 14b | If applicable, eligibility criteria for sites and for individuals who will deliver the interventions (e.g., surgeons, physiotherapists) | Section 2.7, Data collection and management (staff training) |
| Intervention and comparator | 15a | Intervention and comparator with sufficient details to allow replication including how, when, and by whom they will be administered. If relevant, where additional materials describing the intervention and comparator (e.g., intervention manual) can be accessed | Sections 2.2.1–2.2.4, Interventions |
|  | 15b | Criteria for discontinuing or modifying allocated intervention/comparator for a trial participant (e.g., drug dose change in response to harms, participant request, or improving/worsening disease) | Section 2.2.5, Criteria for discontinuing or modifying allocated interventions |
|  | 15c | Strategies to improve adherence to intervention/comparator protocols, if applicable, and any procedures for monitoring adherence (e.g., drug tablet return, sessions attended) | Section 2.4, Safety assessments; Table 1 |
|  | 15d | Concomitant care that is permitted or prohibited during the trial | Section 2.1.2, Inclusion criteria; Section 2.3, Outcomes and covariates |
| Outcomes | 16 | Primary and secondary outcomes, including the specific measurement variable (e.g., systolic blood pressure), analysis metric (e.g., change from baseline, final value, time to event), method of aggregation (e.g., median, proportion), and time point for each outcome | Section 2.3, Outcomes; Section 2.8, Statistical methods; Table 1 |
| Harms | 17 | How harms are defined and will be assessed (e.g., systematically, non-systematically) | Section 2.4, Safety assessments; Section 2.8.6, Safety analysis; Section 2.10.2, Adverse event reporting and harms; Table 1 |
| Participant timeline | 18 | Time schedule of enrollment, interventions (including any run-ins and washouts), assessments, and visits for participants. A schematic diagram is highly recommended (see Figure) | Figure 1; Table 1 |
| Sample size | 19 | How sample size was determined, including all assumptions supporting the sample size calculation | Section 2.5, Sample size |
| Recruitment | 20 | Strategies for achieving adequate participant enrollment to reach target sample size | Section 2.1.1, Recruitment |
| **Methods: Assignment of interventions** | | |  |
| Randomization: |  |  |  |
| Sequence generation | 21a | Who will generate the random allocation sequence and the method used | Section 2.6.1, Sequence generation |
|  | 21b | Type of randomization (simple or restricted) and details of any factors for stratification. To reduce predictability of a random sequence, other details of any planned restriction (e.g., blocking) should be provided in a separate document that is unavailable to those who enroll participants or assign interventions | Section 2.6.1, Sequence generation |
| Allocation concealment  mechanism | 22 | Mechanism used to implement the random allocation sequence (e.g., central computer/telephone; sequentially numbered, opaque, sealed containers), describing any steps to conceal the sequence until interventions are assigned | Section 2.6.2, Allocation concealment |
| Implementation | 23 | Whether the personnel who will enroll and those who will assign participants to the interventions will have access to the random allocation sequence | Sections 2.1.1 and 2.6, Recruitment and assignment of interventions |
| Blinding | 24a | Who will be blinded after assignment to interventions (e.g., participants, care providers, outcome assessors, data analysts) | Section 2.6.3, Blinding |
|  | 24b | If blinded, how blinding will be achieved and description of the similarity of interventions | Sections 2.2.3 and 2.6.3, Sham tTIS and blinding |
|  | 24c | If blinded, circumstances under which unblinding is permissible, and procedure for revealing a participant’s allocated intervention during the trial | N/A |
| **Methods: Data collection, management, and analysis** | | |  |
| Data collection methods | 25a | Plans for assessment and collection of trial data, including any related processes to promote data quality (e.g., duplicate measurements, training of assessors) and a description of trial instruments (e.g., questionnaires, laboratory tests) along with their reliability and validity, if known. Reference to where data collection forms can be accessed, if not in the protocol | Section 2.3, Outcomes; Section 2.7, Data collection and management |
|  | 25b | Plans to promote participant retention and complete follow-up, including list of any outcome data to be collected for participants who discontinue or deviate from intervention protocols | Section 2.2.5, Criteria for discontinuing or modifying allocated interventions |
| Data management | 26 | Plans for data entry, coding, security, and storage, including any related processes to promote data quality (e.g., double data entry; range checks for data values). Reference to where details of data management procedures can be accessed, if not in the protocol | Section 2.7, Data collection and management |
| Statistical methods | 27a | Statistical methods used to compare groups for primary and secondary outcomes, including harms | Section 2.8, Statistical methods; Sections 2.8.1–2.8.6 |
|  | 27b | Definition of who will be included in each analysis (e.g., all randomized participants), and in which group | Section 2.8.1, Primary outcome analysis |
|  | 27c | How missing data will be handled in the analysis | Section 2.8.4, Missing data and sensitivity analyses |
|  | 27d | Methods for any additional analyses (e.g., subgroup and sensitivity analyses) | Sections 2.8.4–2.8.5, Sensitivity and exploratory analyses |
| **Methods: Monitoring** | | |  |
| Data monitoring committee | 28a | Composition of data monitoring committee (DMC); summary of its role and reporting structure; statement of whether it is independent from the sponsor and funder; conflicts of interest and reference to where further details about its charter can be found, if not in the protocol. Alternatively, an explanation of why a DMC is not needed | Section 2.10.1, Composition of the data and safety monitoring board, its role, and reporting structure |
|  | 28b | Explanation of any interim analyses and stopping guidelines, including who will have access to these interim results and make the final decision to terminate the trial | Section 2.10.1, Composition of the data and safety monitoring board, its role, and reporting structure |
| Trial monitoring | 29 | Frequency and procedures for monitoring trial conduct. If there is no monitoring, give explanation | Section 2.10.3, Monitoring of trial conduct |
| **Ethics** | | |  |
| Research ethics approval | 30 | Plans for seeking research ethics committee/institutional review board approval | Section 2.1, Trial design; Ethics statement |
| Protocol amendments | 31 | Plans for communicating important protocol modifications to relevant parties | Section 2.10.3, Monitoring of trial conduct |
| Consent or assent | 32a | Who will obtain informed consent or assent from potential trial participants or authorized proxies, and how | Section 2.1, Trial design; Section 2.1.2, Inclusion criteria; Ethics statement |
|  | 32b | Additional consent provisions for collection and use of participant data and biological specimens in ancillary studies, if applicable | N/A |
| Confidentiality | 33 | How personal information about potential and enrolled participants will be collected, shared, and maintained in order to protect confidentiality before, during, and after the trial | Section 2.7, Data collection and management; Section 2.9, Data sharing; Data availability statement |
| Ancillary and post-trial care | 34 | Provisions, if any, for ancillary and post-trial care, and for compensation to those who suffer harm from trial participation | Section 2.2.5, Criteria for discontinuing or modifying allocated interventions |

Note: N/A = not applicable

Citation: Chan A-W, Boutron I, Hopewell S, Moher D, Schulz KF, et al. SPIRIT 2025 statement: updated guideline for protocols of randomised trials. BMJ 2025;389:e081477. <https://dx.doi.org/10.1136/bmj-2024-081477>
